# Diversity of pneumococcal surface protein A (PspA) among prevalent clones in Spain

**DOI:** 10.1186/1471-2180-9-80

**Published:** 2009-05-06

**Authors:** Dora Rolo, Carmen Ardanuy, Ana Fleites, Rogelio Martín, Josefina Liñares

**Affiliations:** 1 Microbiology Department, Hospital Universitari de Bellvitge, Universitat de Barcelona, IDIBELL, Feixa Llarga s/n, 08907 L'Hospitalet de Llobregat, Barcelona, Spain; 2 CIBERES (CIBER de Enfermedades Respiratorias), ISCIII, Madrid, Spain; 3 Microbiology Department, Hospital Central de Asturias, Oviedo, Spain

## Abstract

**Background:**

PspA is recognized as a major pneumococcal virulence factor and a possible vaccine candidate. The aim of this study was to analyze the PspA family and clade distribution among 112 Spanish pneumococci representatives of dominant clones among patients with invasive disease (n = 66) and nasopharyngeal healthy carriage in children (n = 46).

**Results:**

PspA family 2 was predominant among invasive (63.6%) and carriage (54.3%) pneumococcal isolates. No PspA family 3 isolates were detected and only one strain was PspA negative. Although four clonal complexes contained strains of different clades, a clear association between clade and multi locus sequence typing results was found. Clades 1, 3 and 4 were associated with a wide variety of sequence types (ST) related to multiresistant and antibiotic-susceptible worldwide-disseminated clones. Clade 1 was associated with Spain^6B^-ST90, Spain^14^-ST18, Colombia^5^-ST289, Sweden^1^-ST306, Denmark^14^-ST230 and Sweden^1^-ST304 clones. Clade 3 was associated with Spain^23F^-ST81, Spain^9V^-ST156, Tennessee^14^-ST67, Netherlands^3^-ST180 and Netherlands^7F^-ST191 clones. Clade 4 was related to Sweden^15A^-ST63, Netherlands^18C^-ST113 and Greece^21^-ST193 clones. In contrast, PspA clade was not related to serotype, age or clinical origin of the isolates.

**Conclusion:**

PspA clades were associated with genotypes. PspA family 2 and family 1 were dominant among major Spanish pneumococcal clones isolated from patients with invasive disease and nasopharyngeal carriage in children.

## Background

*Streptococcus pneumoniae *is a major cause of serious community-acquired diseases (such as pneumonia, bacteremia or meningitis), especially in children, the elderly, and among patients with immunological disorders [1]. Nasopharyngeal colonization by *S. pneumoniae *is highly common, particularly among children attending day-care centers and in adults in long-term institutions [2].

Pneumococci are presently divided into 91 serotypes, which are defined by differences in their polysaccharide capsule [3,4]. Two serotype-based vaccines are currently available: the 23-valent polysaccharide vaccine (23V-PSV) which has been shown to be effective in the elderly [5-7], and the heptavalent pneumococcal conjugate vaccine (PCV7) which is used in children below the age of two [5]. In the USA the introduction of PCV7 in children was associated with a decrease in the incidence of invasive pneumococcal diseases (IPD) among children and adults [8]. Moreover, a decrease in IPD caused by resistant strains has also been observed [9]. Although current IPD rates are lower than those observed in the pre-vaccine period, recent reports have shown an increase in IPD caused by non-vaccine serotypes in the USA [10]. In Spain, since the introduction of PCV7, IPD rates due to PCV7 serotypes have decreased in both children and adults, but this improvement has been counterbalanced by an increase in IPD due to non-PCV7 serotypes [11,12].

Currently, two new conjugated vaccines are under development – 10-valent and the 13-valent vaccines, which both contain some emerging serotypes [13]. Alternative vaccines are also being evaluated, such as those based on pneumococcal virulence proteins. Many pneumococcal proteins have been investigated as vaccine candidates, for instance, pneumolysin, PsaA, PspC, and PspA [13,14]. The pneumococcal surface protein A (PspA) is an important virulence factor which interferes with complement deposition on the pneumococcal surface [15] and is detected in almost all pneumococci [16-18]. It is highly immunogenic and protective and has proved to be highly cross-reactive both in various animal models [15,19,20] and in humans [21]. It is hypothesized that a PspA-based vaccine could protect against invasive disease and also eliminate the carrier state [15-22]. PspA is constituted by five domains: a signal peptide, a α-helical charged domain which includes a clade-defining region, a proline-rich region, a choline-binding domain and a C-terminal domain [16]. Although the PspA encoding gene (*psp*A) is highly genetically variable, the classification by families is based on nucleotide and amino acid identity. Each of the three PspA families is subdivided into different clades: family 1 is composed by two clades (clade 1 and 2), family 2 comprises three clades (clades 3, 4 and 5), and PspA family 3 has only one divergent clade (clade 6) [16].

The aim of this study was to analyze the distribution of the PspA clades among a pneumococcal collection representative of major clones found in two previous studies among healthy children carriers [23] and patients with invasive disease [11].

## Methods

### Bacterial strains

One hundred and twelve pneumococcal strains previously characterized by pulsed field gel electrophoresis (PFGE) with *Sma*I restriction enzyme, as described elsewhere [24] and serotyped by Quellung reaction [25], were selected as follows:

a) Forty-nine pneumococci isolated from adults with IPD in Barcelona (NorthEast of Spain) between 1997 and 2007 (Additional file 1). These 49 strains were representative of the 32 major genotypes found among 968 pneumococci causing IPD in adult patients in Barcelona [11]. The pneumococcal strains selected were isolated from blood (n = 36), CSF (n = 11) and other sterile fluids (n = 2);

b) Seventeen pneumococci isolated from patients with IPD in Oviedo (Northern Spain) in 2004–2005 (Additional file 1) selected as representative of the nine major PFGE patterns among 101 consecutive invasive strains (unpublished data). The selected strains were isolated from blood (n = 11), CSF (n = 3) and other sterile fluids (n = 3);

c) Forty-six pneumococci were selected from nasopharyngeal carriers aged from 1 to 4 years old, in Oviedo (Northern Spain) in 2004–2005 [23] (Additional file 1). These strains were representative of 29 dominant PFGE patterns found among 365 pneumococci isolated from children attending 23 day-care centers.

### Antimicrobial susceptibility testing

The minimal inhibitory concentration (MIC) was determined by microdilution following CLSI guidelines [26] using a panel of antimicrobials which included penicillin, erythromycin, clindamycin, tetracycline, chloramphenicol and cotrimoxazol. Resistant strains were defined according to CLSI criteria [27]. *S. pneumoniae *ATCC 49619 was used as control.

### Multilocus sequence typing (MLST) and eBURST

MLST was performed as described previously [28]. The allele's number and sequence types (ST) were assigned using the pneumococcal MLST website [29]. Lineage assignment was achieved by eBURST analysis [30,31].

### PspA detection

**Figure 1:**
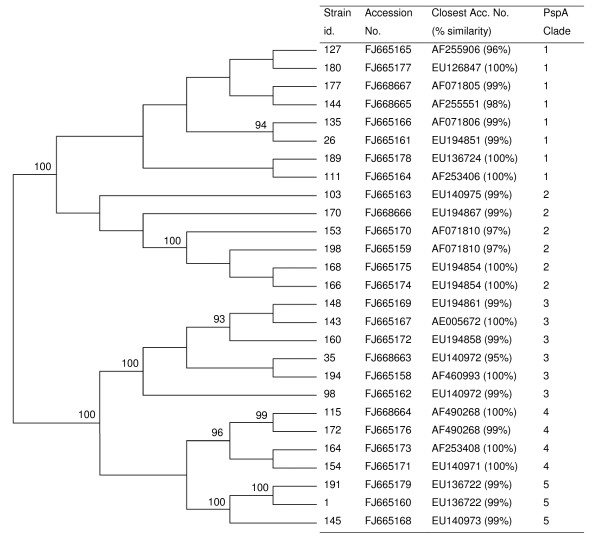
**Phylogenetic tree of a 373-bp region that includes *psp*A clade-defining region**. Phylogenetic and molecular evolutionary analyses were conducted with the MEGA4 program (version 4.1) [36] by the Neighbor-Joining method. Only bootstrap confidence intervals exceeding 90% are shown.

The PCRs were carried out in a standard PCR mixture of 50 μl containing 2.5 mM of MgCl_2_, 240 μM (each) of deoxynucleoside triphosphates (dNTPs), 0.3 μM of each primer, and 2 U of *Taq *DNA polymerase (AmpliTaq Gold^®^, Roche). The cycle conditions consisted of: an initial 94°C (10 min), 30 cycles of 94°C (1 min), 55°C (1 min) and 72°C (3 min), followed by 72°C (10 min). A multiplex PCR reaction was tested [32], but some samples did not amplify with LSM12/SKH63 [32,33] or LSM12/SKH52 [22] primer combinations. The combination of LSM12/SKH2 primers [16] was successfully used for all samples except one. The isolate that did not amplify was retested with the same cycle pattern at an annealing temperature of 52°C and with different primer combinations (LSM12/SKH63, LSM12/SKH52 and LSM12/SKH2). Controls for PspA family 1 (Spain^14^-ST18) and PspA family 2 (Spain^23F^-ST81) were run in each reaction set. PCR products were purified and sequenced using SKH2 primer, as described elsewhere [34]. Sequence edition was performed using the SeqScape version 2.1.1 (Applied Biosystems) software, while DNA sequences were assigned using BLAST [35]. Clade type was established when the closest match presented identity higher than 95% (Figure 1). The phylogenetic and molecular evolutionary analyses were conducted using MEGA4 version 4.1 software [36]. The evolutionary history was inferred using the Neighbor-Joining method and the bootstrap consensus tree inferred from 1000 replicates. The evolutionary distances were computed using the Kimura 2-parameter method [36].

### Nucleotide sequence accession numbers

The sequences of 27 isolates representing all the different sequences from this collection were deposited in GenBank with accession numbers: [GenBank:FJ665158, GenBank:FJ665159, GenBank:FJ665160, GenBank:FJ665161, GenBank:FJ665162, GenBank:FJ665163, GenBank:FJ665164, GenBank:FJ665165, GenBank:FJ665166, GenBank:FJ665167, GenBank:FJ665168, GenBank:FJ665169, GenBank:FJ665170, GenBank:FJ665171, GenBank:FJ665172, GenBank:FJ665173, GenBank:FJ665174, GenBank:FJ665175, GenBank:FJ665176, GenBank:FJ665177, GenBank:FJ665178, GenBank:FJ665179, GenBank:FJ668663, GenBank:FJ668664, GenBank:FJ668665, GenBank:FJ668666, GenBank:FJ668667].

## Results and discussion

### PspA families and clade distribution

Among the 112 pneumococci studied, the majority (59.8%, 67/112) were identified as belonging to PspA family 2 (31 isolates of clade 3, 27 of clade 4 and nine of clade 5), while the remaining 39.3% (44/112) belonged to family 1 (29 isolates of clade 1 and 15 of clade 2). One strain was negative. No PspA family 3 isolates were detected. Figure 1 shows the phylogenetic tree of the 27 new PspA sequences found as well as the accession numbers and the percentage of identity to previously published sequences. Sequences of strains of PspA families 1 and 2 were precisely grouped, and all were joined into their respective clades. The similarity of isolates of the same family ranged from 84% to 100%. The percentage of similarity within isolates of the same clade ranged as follows: clade 1 (84 to 95), clade 2 (84 to 100), clade 3 (93 to 99), clade 4 (91 to 98) and clade 5 (96 to 100).

Among the 66 pneumococci isolated from patients with IPD, 63,6% (42/66) were found to be of PspA family 2 (24 isolates of clade 3, 12 of clade 4 and six of clade 5), 34.8% (23/66) of family 1 (20 isolates of clade 1 and three of clade 2) and one isolate was negative. The high prevalence of PspA family 2 among pneumococci isolated from adults with IPD has already been reported in Spain, Canada, Sweden, the USA and France [37,38], although in Australia, the UK and Japan PspA family 1 was the most prevalent [38,39]. The dominance of family 2, clade 3 observed in our study has also been reported in other studies of pneumococci causing IPD in adults in France [37] and in children from Germany [40].

PspA family 2 was also dominant (54.3%, 25/46) among pneumococci isolated from the nasopharynx of healthy children (seven of clade 3, 15 of clade 4 and three of clade 5), while family 1 accounted for 45.7% (21/46) of the strains (nine of clade 1 and 12 of clade 2). These data are in agreement with two PspA studies [32,34] which found PspA family 2 to be dominant among pneumococci isolated from Brazilian children carriers. Moreover, the clade distribution also showed a prevalence of clade 4, followed by clade 1 and clade 3 [34]. A recent publication with data collected from pneumococci isolated from nasopharyngeal carriage in Finnish children showed similar prevalences of PspA family 1 and family 2 [41].

Additional file 1 shows the results for PspA family and clade of the 112 pneumococcal isolates studied with respect to sequence type (ST), serotype, resistance pattern and source. The pneumococci isolated from children carriers or from patients with IPD invasive disease seem to be indistinct, suggesting that PspA type is independent of age or clinical origin, as has been shown elsewhere [32,34].

### Relationship between PspA and serotypes

In agreement with previous studies [16,32,42] our results showed that PspA clades are independent of serotypes. Pneumococci of the same serotype were associated with different PspA clades from the same or a different family (Additional file 1). For instance, pneumococci of serotype 6A could have PspA clade 2 (family 1), whereas pneumococci of serotype 6B could express PspA clades 1, 2, 4 or 5 (families 1 and 2). Since PspA is independent of serotype, PspA-based vaccines could improve upon the results obtained with serotype-based vaccines and might avoid a possible serotype replacement, as previously observed [10]. Since a PspA-based vaccine potentially has high coverage due to the fact that it is cross protective and immunogenic among children and adults [21], similar data should be investigated in other geographical areas in order to study the potential coverage of a PspA-based vaccine, and to adapt it to different formulations if necessary.

### Relationship between PspA and clones

PspA clade classification was related to genotypes, and all strains with the same ST always presented the same PspA clade (see Additional file 1), regardless of origin or capsular type. In spite of the high genetical variability of *psp*A gene, all isolates of the same ST showed 100% of identity between their sequences. For instance, among nine pneumococci with ST63 obtained from invasive and carriage samples, four capsular types were found (15A, 19A, 19F and 23F) but all of them had PspA of clade 4 (see Additional file 1). However, other authors have found different PspA families among isolates that shared a common ST [41]. In our study, among 65 STs found, only 7 accounted for more than three isolates (ST63 n = 9, ST156 n = 5, and ST42, ST260, ST180, ST62 and ST81 with four isolates each). This fact may be a limitation of the present study and may affect its capacity to assess the relationship between ST and PspA.

The eBURST analysis reveals the presence of 15 clonal complexes (CC) and 22 singletons (S) (Additional file 1). The association of CC and S with clade was as follows: clade 1 (23 STs: 7 CC and 7 S), clade 2 (11 STs: 4 CC and 2 S), clade 3 (14 STs: 3 CC and 6 S), clade 4 (13 STs: 4 CC and 4 S), and clade 5 (4 STs: 1 CC and 3 S). Four CCs contained only clade 1-associated STs, three CCs contained clade 4-related STs, two CCs contained only clade 2-related STs, and two CC contained clade 3-related STs. Four CCs contained STs related to two different clades of the same or a different PspA family.

The relationship of PspA clade with multiresistant worldwide-disseminated clones described by the Pneumococcal Molecular Epidemiology Network (PMEN) [24] has been reported previously [32,42-44]. Our study provides further information since the majority of CCs found are related to PMEN clones. For instance, the Spain^9V^-ST156 (CC156) clone, which is one of the most important clones causing IPD worldwide [11,32,42,43], included six STs in the present study. All six STs of this CC had PspA clade 3, suggesting that PspA is highly conserved in this clone, even in SLV or DLV or when expressing capsular type 9 V or 14. Similar results were found among other CCs related to other multiresistant PMEN clones: Spain^6B^-ST90 (clade 1), Spain^14^-ST18 (clade 1), Denmark^14^-ST230 (clade 1), Spain^23F^-ST81 (clade 3), Greece^21^-ST193 (clade 4) and Sweden^15A^-ST63 (clade 4). The CC439 related to PMEN clone Tennessee^23F^-ST37, which included six STs in our study, had two PspA clades (1 and 4). This finding was in agreement with a study from Finland, which found PspA from families 1 and 2 among isolates within the same or different ST of this CC439 [41].

There is still little information about the relationship between PspA clade and antibiotic-susceptible PMEN clones, since the available data only refer to family level [42]. Our study provides new information about the antibiotic-susceptible clones, which are associated with the increase of IPD observed in recent years in some European countries [11,45] and in the USA [10]. For instance, the Sweden^1^-ST306 clone had clade 1. This clone has been described as the cause of IPD outbreaks in Europe and its frequency is currently increasing in Spain as cause of IPD and, especially, parapneumonic empyema in children [45]. CCs which were also related to antibiotic-susceptible PMEN clones included clade 1 (Colombia^5^-ST289 and Sweden^1^-ST304) and clade 3 (Netherlands^7F^-ST191, Netherlands^3^-ST180 and Tennessee^14^-ST67). Other associations of PspA clade with emerging clones were also observed such as clade 1 for serotype 22-ST433 and serotype 10A-CC97, and clade 5 for serotype 12-ST989. The CC53 (Netherlands^8^-ST53) included strains of two clades: clade 1 for those isolated with ST53 that were serotype 8, and clade 3 for isolates with ST62 (DLV) that were serotype 11A or non-typeable.

Since PspA type is associated with genotype, and with our knowledge of the clonal distribution of pneumococci causing IPD in Southern Barcelona area [11] we estimate that at least 45.1% would be of PspA family 2, and 23.4% of family 1. The most prevalent clades among invasive pneumococci would be clade 3 (48.2%) and clade 1 (33.7%). Similarly, we estimate that among the pneumococci isolated from children carriage [23] at least 31.6% appear to be PspA family 2 and 29.8% PspA family 1, with clade 3 (26.0%) and clade 1 (22.5%) being the most frequent.

## Conclusion

Our study supports previous data [34,38,39,43] demonstrating that PspA family and clade distribution are independent of serotype, age and clinical origin of the isolates, but are highly associated with genotype. This study suggests that PspA family 1 and 2 molecules should be included in future PspA-based vaccine formulations. Further studies are needed to determine the genetic diversity of PspA in each geographical area.

## Authors' contributions

CA and JL conceived the study and participated in its design. AF, RM and JL participated in field and clinical aspects of the study. DR and CA carried out the molecular genetic studies and sequence alignment. DR and CA wrote the manuscript, which was coordinated and critically reviewed by JL. All authors read and approved the final manuscript.

## Supplementary Material

Additional File 1 **Table 1**. Characteristics of 112 representative pneumococcal strains selected for this study. Click here for file
