# Supplementary material for: Diversity of pneumococcal surface protein A (PspA) among prevalent clones in Spain
Source: BMC Microbiol. 2009 May 6;9:80. doi: 10.1186/1471-2180-9-80 (PMC2684541; doi:10.1186/1471-2180-9-80)
Supplement: Additional File 1 — Table 1. Characteristics of 112 representative pneumococcal strains selected for this study. [file 1471-2180-9-80-S1.doc]

**Tables**

**Table 1 - Characteristics of 112 representative pneumococcal strains selected for this study.**

| PspA  Family | PspA Clade | Strain id. | PFGE  Pattern | Clonal Complex^a^ | ST^b^ | Related PMEN clone^c^ | Serotype^d^ | Susceptibility Pattern^e^ | Source^f^ | Age (years) | Isolation Place^g^ | Accession Number |
| --- | --- | --- | --- | --- | --- | --- | --- | --- | --- | --- | --- | --- |
| Family 1 | Clade 1 | 111 | C-9 | CC9 | 9 | England^14^-ST9 | 14 | E | Pharynx | 2 | OVD | FJ665164 |
|  |  | 19 | C-9 | CC9 | 1817 | England^14^-ST9 (SLV) | 14 | E | CSF | 30 | BCN | FJ665164 |
|  |  | 180 | PA | CC439 | 439 | Tennessee^23F^-ST37 (SLV) | 23B | Susceptible | Pharynx | 3 | OVD | FJ665177 |
|  |  | 183 | PB | CC439 | 33 | Tennessee^23F^-ST37 (SLV) | 23F | Susceptible | Pharynx | 2 | OVD | FJ665177 |
|  |  | 127 | PC | CC439 | 311 | Tennessee^23F^-ST37 (DLV) | 23F | Susceptible | Blood | 77 | BCN | FJ665165 |
|  |  | 181 | PD | CC439 | 36 |  | 23F | Susceptible | Pharynx | 4 | OVD | FJ665165 |
|  |  | 114 | I | CC53 | 53 | Netherlands^8^-ST53 | 8 | Susceptible | Blood | 65 | BCN | FJ668665 |
|  |  | 144 | I | CC53 | 53 | Netherlands^8^-ST53 | 8 | Susceptible | Blood | 70 | OVD | FJ668665 |
|  |  | 135 | C-2 | CC90 | 90 | Spain^6B^-ST90 | 6B | PEClCTSxT | Blood | 78 | OVD | FJ665166 |
|  |  | 146 | C-2 | CC90 | 96 | Spain^6B^-ST90 (SLV) | 6B | PEClCTSxT | Pharynx | 2 | OVD | FJ665166 |
|  |  | 11 | C-2 | CC90 | 1624 | Spain^6B^-ST90 (SLV) | 6B | PEClTSxT | Pharynx | 2 | OVD | FJ665166 |
|  |  | 123 | L | CC97 | 97 |  | 10A | Susceptible | Blood | 71 | BCN | FJ665165 |
|  |  | 159 | L | CC97 | 97 |  | 10A | Susceptible | Pharynx | 2 | OVD | FJ665165 |
|  |  | 195 | L | CC97 | 1282 |  | 10A | ECl | Blood | 89 | BCN | FJ665165 |
|  |  | 190 | L | CC97 | 3258^a^ |  | 10A | Susceptible | Blood | 43 | BCN | FJ665165 |
|  |  | 110 | Z | CC230 | 276 | Denmark^14^-ST230 (SLV) | 19A | PEClTSxT | Pharynx | 2 | OVD | FJ665165 |
|  |  | 178 | Z | CC230 | 276 | Denmark^14^-ST230 (SLV) | 19A | PEClTSxT | Pharynx | 2 | OVD | FJ665165 |
|  |  | 118 | C-19 | CC289 | 289 | Colombia^5^-ST289 | 5 | Susceptible | Blood | 43 | BCN | FJ665178 |
|  |  | 189 | C-19 | CC289 | 1223 | Colombia^5^-ST289 (SLV) | 5 | Susceptible | Blood | 83 | BCN | FJ665178 |
|  |  | 20 | C-5 | S | 18 | Spain^14^-ST18 | 14 | PEClTCSxT | Blood | 67 | BCN | FJ665164 |
|  |  | 24 | E | S | 306 | Sweden^1^-ST306 | 1 | Susceptible | P. fluid | 87 | BCN | FJ665161 |
|  |  | 25 | E | S | 306 | Sweden^1^-ST306 | 1 | Susceptible | Blood | 51 | BCN | FJ665161 |
|  |  | 26 | F | S | 304 | Sweden^1^-ST304 | 1 | Susceptible | Blood | 67 | BCN | FJ665161 |
|  |  | 27 | F | S | 304 | Sweden^1^-ST304 | 1 | Susceptible | Blood | 78 | BCN | FJ665161 |
|  |  | 177 | PJ | S | 2730 |  | 9V | SxT | Pharynx | 3 | OVD | FJ668667 |
|  |  | 124 | M | S | 1026 |  | 20 | Susceptible | Blood | 71 | BCN | FJ665165 |
|  |  | 187 | Q | S | 1372 |  | 22F | Susceptible | CSF | 41 | BCN | FJ665177 |
|  |  | 141 | Q | S | 433 |  | 22F | Susceptible | Blood | 50 | OVD | FJ665165 |
|  |  | 188 | Q | S | 433 |  | 22F | Susceptible | Blood | 72 | BCN | FJ665165 |
|  | Clade 2 | 153 | C-9 | CC9 | 1964 | England^14^-ST9 (DLV) | 14 | PEClSxT | Pharynx | 3 | OVD | FJ665170 |
|  |  | 39 | C | CC88 | 88 |  | 19F | PEClTCSxT | Blood | 81 | OVD | FJ665163 |
|  |  | 103 | C | CC88 | 87 |  | 19F | PTC | Blood | 84 | OVD | FJ665163 |
|  |  | 165 | C | CC88 | 2103 |  | 19F | PSxT | Pharynx | 2 | OVD | FJ665163 |
|  |  | 179 | C | CC88 | 2103 |  | 19F | PSxT | Pharynx | 2 | OVD | FJ665163 |
|  |  | 168 | C-20 | CC315 | 386 | Poland^6B^-ST315 (DLV) | 6A | PEClTSxT | Pharynx | 1 | OVD | FJ665175 |
|  |  | 169 | PE | CC473 | 473 |  | 6B | PE | Pharynx | 2 | OVD | FJ668666 |
|  |  | 170 | PE | CC473 | 473 |  | 6A | PE | Pharynx | 2 | OVD | FJ668666 |
|  |  | 173 | PF | CC473 | 471 |  | 6B | PE | Pharynx | 2 | OVD | FJ668666 |
|  |  | 175 | PG | CC473 | 471 |  | 6A | PE | Pharynx | 3 | OVD | FJ668666 |
|  |  | 176 | PG | CC473 | 471 |  | 6B | PE | Pharynx | 3 | OVD | FJ668666 |
|  |  | 171 | PH | CC473 | 1876 |  | 6A | PE | Pharynx | 1 | OVD | FJ668666 |
|  |  | 174 | PI | CC473 | 3257^a^ |  | 6A | PE | Pharynx | 2 | OVD | FJ668666 |
|  |  | 166 | C-21 | S | 179 | Portugal^19F^-ST177 (SLV) | 19F | EClT | Pharynx | 3 | OVD | FJ665174 |
|  |  | 198 | R | S | 2217 |  | 35F | Susceptible | CSF | 35 | BCN | FJ665159 |
| Family 2 | Clade 3 | 30 | H | CC53 | 62 | Netherlands^8^-ST53 (DLV) | 11A | SxT | Blood | 30 | BCN | FJ665172 |
|  |  | 31 | H | CC53 | 62 | Netherlands^8^-ST53 (DLV) | 11A | SxT | Blood | 38 | BCN | FJ665172 |
|  |  | 160 | H | CC53 | 62 | Netherlands^8^-ST53 (DLV) | 11A | SxT | Pharynx | 3 | OVD | FJ665172 |
|  |  | 161 | H | CC53 | 62 | Netherlands^8^-ST53 (DLV) | NT | SxT | Pharynx | 3 | OVD | FJ665172 |
|  |  | 21 | C-3 | CC156 | 156 | Spain^9V^-ST156 | 14 | PSxT | Blood | 75 | BCN | FJ665169 |
|  |  | 100 | C-3 | CC156 | 156 | Spain^9V^-ST156 | 14 | PSxT | CSF | <1 | OVD | FJ665169 |
|  |  | 136 | C-3 | CC156 | 156 | Spain^9V^-ST156 | 14 | PSxT | P. fluid | 2 | OVD | FJ665169 |
|  |  | 137 | C-3 | CC156 | 156 | Spain^9V^-ST156 | 9V | PSxT | CSF | 77 | OVD | FJ665169 |
|  |  | 148 | C-3 | CC156 | 156 | Spain^9V^-ST156 | 19F | PSxT | Pharynx | 2 | OVD | FJ665169 |
|  |  | 147 | C-3 | CC156 | 162 | Spain^9V^-ST156 (SLV) | 9V | EClTSxT | Pharynx | 2 | OVD | FJ665169 |
|  |  | 17 | C-3 | CC156 | 838 | Spain^9V^-ST156 (SLV) | 9V | PSxT | Blood | 43 | BCN | FJ665169 |
|  |  | 18 | C-3 | CC156 | 2587 | Spain^9V^-ST156 (DLV) | 9V | PSxT | Blood | 91 | BCN | FJ665169 |
|  |  | 149 | C-3 | CC156 | 3707^a^ | Spain^9V^-ST156 (DLV) | 14 | PSxT | Pharynx | 4 | OVD | FJ665169 |
|  |  | 151 | C-3 | CC156 | 2828^a^ | Spain^9V^-ST156 (DLV) | 14 | PEClCTSxT | Pharynx | 3 | OVD | FJ665169 |
|  |  | 43 | C-1 | CC81 | 81 | Spain^23F^-ST81 | 23F | PEClTCSxT | Blood | 93 | BCN | FJ665162 |
|  |  | 126 | C-1 | CC81 | 81 | Spain^23F^-ST81 | 23F | PEClTCSxT | CSF | 62 | BCN | FJ665162 |
|  |  | 98 | C-1 | CC81 | 81 | Spain^23F^-ST81 | 23F | PEClTCSxT | Pharynx | 2 | OVD | FJ665162 |
|  |  | 134 | C-1 | CC81 | 81 | Spain^23F^-ST81 | 23F | PEClTCSxT | Blood | 69 | OVD | FJ665162 |
|  |  | 42 | O | S | 202 | Taiwan^19F^-ST236 (DLV) | 19A | EClTSxT | Blood | 36 | BCN | FJ665167 |
|  |  | 109 | O | S | 202 | Taiwan^19F^-ST236 (DLV) | 19A | EClTSxT | P. fluid | 2 | OVD | FJ665167 |
|  |  | 196 | S | S | 67 | Tennessee^14^-ST67 | 9N | Susceptible | Blood | 79 | BCN | FJ665167 |
|  |  | 197 | S | S | 67 | Tennessee^14^-ST67 | 9N | Susceptible | CSF | 57 | BCN | FJ665167 |
|  |  | 6 | B | S | 180 | Netherlands^3^-ST180 | 3 | Susceptible | Blood | 70 | BCN | FJ668663 |
|  |  | 35 | B | S | 180 | Netherlands^3^-ST180 | 3 | Susceptible | Blood | 57 | BCN | FJ668663 |
|  |  | 125 | B | S | 180 | Netherlands^3^-ST180 | 3 | Susceptible | CSF | 22 | BCN | FJ668663 |
|  |  | 140 | B | S | 180 | Netherlands^3^-ST180 | 3 | Susceptible | Blood | 65 | OVD | FJ668663 |
|  |  | 120 | J | S | 191 | Netherlands^7F^-ST191 | 7F | Susceptible | Blood | 25 | BCN | FJ665169 |
|  |  | 121 | J | S | 191 | Netherlands^7F^-ST191 | 7F | Susceptible | CSF | 61 | BCN | FJ665169 |
|  |  | 28 | G | S | 247 |  | 4 | Susceptible | Blood | 65 | BCN | FJ665167 |
|  |  | 143 | G | S | 247 |  | 4 | Susceptible | Blood | 81 | OVD | FJ665167 |
|  |  | 194 | T | S | 30 |  | 16 | EClTCSxT | Blood | 68 | BCN | FJ665158 |
|  | Clade 4 | 112 | Y | CC439 | 42 | Tennessee^23F^-ST37 (DLV) | 23A | EClTCSxT | CSF | 37 | BCN | FJ665171 |
|  |  | 108 | Y | CC439 | 42 | Tennessee^23F^-ST37 (DLV) | 23A | EClTCSxT | Aq. Humor | 1 | OVD | FJ665171 |
|  |  | 185 | Y | CC439 | 42 | Tennessee^23F^-ST37 (DLV) | 23B | EClTCSxT | Pharynx | 4 | OVD | FJ665171 |
|  |  | 186 | Y | CC439 | 42 | Tennessee^23F^-ST37 (DLV) | 23A | EClTCSxT | Pharynx | 2 | OVD | FJ665171 |
|  |  | 167 | PK | CC439 | 2829^a^ | Tennessee^23F^-ST37 (DLV) | 23A | Susceptible | Pharynx | 2 | OVD | FJ665171 |
|  |  | 119 | C-25 | CC63 | 63 | Sweden^15A^-ST63 | 15A | PEClT | Blood | 62 | BCN | FJ665171 |
|  |  | 157 | C-25 | CC63 | 63 | Sweden^15A^-ST63 | 15A | PEClT | Pharynx | 4 | OVD | FJ665171 |
|  |  | 139 | C-25 | CC63 | 63 | Sweden^15A^-ST63 | 19F | PEClT | CSF | 45 | OVD | FJ665171 |
|  |  | 155 | C-25 | CC63 | 63 | Sweden^15A^-ST63 | 19F | PEClT | Pharynx | 2 | OVD | FJ665171 |
|  |  | 138 | C-25 | CC63 | 63 | Sweden^15A^-ST63 | 19A | PEClT | Blood | 2 | OVD | FJ665171 |
|  |  | 105 | C-25 | CC63 | 63 | Sweden^15A^-ST63 | 19A | PEClT | Pharynx | 3 | OVD | FJ665171 |
|  |  | 154 | C-25 | CC63 | 63 | Sweden^15A^-ST63 | 19A | PEClT | Pharynx | 2 | OVD | FJ665171 |
|  |  | 156 | C-25 | CC63 | 63 | Sweden^15A^-ST63 | 23F | PEClT | Pharynx | 3 | OVD | FJ665171 |
|  |  | 158 | C-25 | CC63 | 63 | Sweden^15A^-ST63 | NT | PEClT | Pharynx | 1 | OVD | FJ665171 |
|  |  | 102 | C-25 | CC63 | 374 | Sweden^15A^-ST63 (SLV) | 15A | PEClT | Blood | 56 | OVD | FJ665171 |
|  |  | 104 | C-25 | CC63 | 2100 | Sweden^15A^-ST63 (SLV) | 19F | PEClT | Blood | 74 | OVD | FJ665171 |
|  |  | 116 | K | CC113 | 113 | Netherlands^18C^-ST113 | 18C | SxT | CSF | 81 | BCN | FJ665171 |
|  |  | 117 | K | CC113 | 110 | Netherlands^18C^-ST113 (SLV) | 18C | SxT | Blood | 85 | BCN | FJ665171 |
|  |  | 150 | Pl | CC193 | 193 | Greece^21^-ST193 | 19A | EClT | Pharynx | 1 | OVD | FJ665176 |
|  |  | 162 | PM | CC193 | 193 | Greece^21^-ST193 | 15B | EClT | Pharynx | 2 | OVD | FJ665176 |
|  |  | 163 | PM | CC193 | 193 | Greece^21^-ST193 | 15C | EClT | Pharynx | 2 | OVD | FJ665176 |
|  |  | 152 | PN | CC193 | 2110 | Greece^21^-ST193 (SLV) | 19A | EClC | Pharynx | 4 | OVD | FJ665176 |
|  |  | 192 | U | S | 558 | Utah^35B^-ST377 (SLV) | 35B | P | Blood | 41 | BCN | FJ665171 |
|  |  | 193 | U | S | 558 | Utah^35B^-ST377 (SLV) | 35B | P | Blood | 88 | BCN | FJ665171 |
|  |  | 172 | PO | S | 894 |  | 6B | PEClTCSxT | Pharynx | 1 | OVD | FJ665176 |
|  |  | 115 | D | S | 2312 |  | 8 | Susceptible | Blood | 19 | BCN | FJ668664 |
|  |  | 164 | PP | S | 392 |  | 17 | Susceptible | Pharynx | 3 | OVD | FJ665173 |
|  | Clade 5 | 122 | C-20 | CC315 | 315 | Poland^6B^-ST315 | 6B | PEClT | Blood | 65 | BCN | FJ665168 |
|  |  | 145 | PQ | S | 338 | Colombia^23F^-ST338 | 23F | PE | Pharynx | 2 | OVD | FJ665168 |
|  |  | 182 | PR | S | 338 | Colombia^23F^-ST338 | 23F | PE | Pharynx | 1 | OVD | FJ665168 |
|  |  | 184 | PS | S | 338 | Colombia^23F^-ST338 | 23F | PE | Pharynx | 1 | OVD | FJ665168 |
|  |  | 8 | A | S | 260 |  | 3 | Susceptible | CSF | 67 | BCN | FJ665160 |
|  |  | 9 | A | S | 260 |  | 3 | Susceptible | Blood | 67 | BCN | FJ665160 |
|  |  | 1 | A | S | 260 |  | 3 | Susceptible | CSF | 63 | BCN | FJ665160 |
|  |  | 142 | A | S | 260 |  | 3 | Susceptible | Blood | 59 | BCN | FJ665160 |
|  |  | 191 | V | S | 989 |  | 12 | C | TNA | 47 | BCN | FJ665179 |
| Negative |  | 113 | I | CC53 | 53 | Netherlands^8^-ST53 | 8 | Susceptible | Blood | 64 | BCN |  |

^a^ CC: Clonal complex, S: singleton; ^b^ New sequence types (ST); ^c^ SLV: Single locus variant, DLV: Double locus variant; ^d^ Underlyned serotypes indicates capsular switches, NT: non-typeable; ^e^ P: Penicillin non-susceptible (MIC ≥0.12μg/mL), E: Erythromycin resistant (MIC ≥0.5μg/mL), Cl: Clindamycin resistant (MIC ≥0.5μg/mL), T: Tetracycline resistant (MIC ≥4μg/mL), C: Chloramphenicol resistant (MIC ≥8μg/mL), SxT: Cotrimoxazole resistant (MIC ≥1/19μg/mL); ^f^ Pharynx: Pharyngeal swabs, CSF: Cerebrospinal fluid, P. fluid: Pleural fluid, Aq. Humor: Aqueous Humor, TNA: Transthoracic Needle Aspiration; ^g^ OVD: Oviedo, BCN: Barcelona.
